# The evolution of the *Aristolochia pallida* complex (Aristolochiaceae) challenges traditional taxonomy and reflects large‐scale glacial refugia in the Mediterranean

**DOI:** 10.1002/ece3.8765

**Published:** 2022-03-31

**Authors:** Cornelia Krause, Birgit Oelschlägel, Hafez Mahfoud, Dominik Frank, Gérard Lecocq, Lulëzim Shuka, Christoph Neinhuis, Pablo Vargas, Aycan Tosunoglu, Mike Thiv, Stefan Wanke

**Affiliations:** ^1^ Botany Department State Museum of Natural History Stuttgart Stuttgart Germany; ^2^ Institut für Botanik Technische Universität Dresden Dresden Germany; ^3^ La Seyne sur Mer France; ^4^ Department of Biology, Faculty of Natural Sciences Tirana University Tiranë Albania; ^5^ Real Jardín Botánico (RJB‐CSIC) Madrid Spain; ^6^ Department of Biology Faculty of Arts and Science Bursa Uludag University Bursa Turkey; ^7^ Departamento de Botánica, Instituto de Biología Universidad Nacional Autónoma de México Mexico City Mexico

**Keywords:** *Aristolochia pallida* group, biogeography, Mediterranean, phylogeny, taxonomy

## Abstract

The taxonomy of the Mediterranean *Aristolochia pallida* complex has been under debate since several decades with the following species currently recognized: *A*. *pallida*, *A*. *lutea*, *A*. *nardiana*, *A*. *microstoma*, *A*. *merxmuelleri*, *A*. *croatica*, and *A*. *castellana*. These taxa are distributed from Iberia to Turkey. To reconstruct phylogenetic and biogeographic patterns, we employed cpDNA sequence variation using both noncoding (intron and spacer) and protein‐coding regions (i.e., *trnK* intron, *matK* gene, and *trnK*‐*psbA* spacer). Our results show that the morphology‐based traditional taxonomy was not corroborated by our phylogenetic analyses. *Aristolochia pallida*, *A*. *lutea*, *A*. *nardiana*, and *A*. *microstoma* were not monophyletic. Instead, strong geographic signals were detected. Two major clades, one exclusively occurring in Greece and a second one of pan‐Mediterranean distribution, were found. Several subclades distributed in Greece, NW Turkey, Italy, as well as amphi‐Adriatic subclades, and a subgroup of southern France and Spain, were revealed. The distribution areas of these groups are in close vicinity to hypothesized glacial refugia areas in the Mediterranean. According to molecular clock analyses the diversification of this complex started around 3–3.3 my, before the onset of glaciation cycles, and the further evolution of and within major lineages falls into the Pleistocene. Based on these data, we conclude that the *Aristolochia pallida* alliance survived in different Mediterranean refugia rarely with low, but often with a high potential for range extension, and a high degree of morphological diversity.

## Graphical abstract

The Mediterranean *Aristolochia pallida* complex (with *A*. *pallida*, *A*. *lutea*, *A*. *nardiana*, *A*. *microstoma*, *A*. *merxmuelleri*, *A*. *croatica* and *A*. *castellana* currently recognized) shows a high degree of morphological diversity. To reconstruct phylogenetic and biogeographic patterns, we employed cpDNA sequence variation using both non‐coding and protein coding regions. Our results show that the morphology‐based traditional taxonomy was not corroborated by our phylogenetic analyses. *Aristolochia pallida*, *A*. *lutea*, *A*. *nardiana* and *A*. *microstoma* were not monophyletic. Instead, strong geographic signals were detected. We found several subclades which correspond well to hypothesised glacial refugia in the Mediterranean.

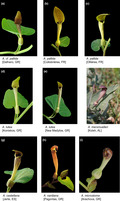

## 1 INTRODUCTION

The Mediterranean is one of the world's biodiversity hotspots (Médail & Diadema, 2009; Medail & Quezel, 1997). It comprises about 25,000 plant species of which 50% are endemic (Cowling et al., 1996). As adaptation to seasonal climates with summer droughts, annual herbs, sclerophyllous shrubs or trees, and geophytes dominate the flora, the taxonomy of some groups is, however, poorly understood. For example, a classic problem is the species delimitation of *Ophrys* (Orchidaceae) which can be morphologically very similar (Véla et al., 2015). Molecular analyses provided helpful data to solve taxonomic questions. Gurushidze et al. (2008) found cryptic species among Mediterranean *Allium* L. (Amaryllidaceae) and Pillon et al. (2006) revealed high genetic diversity in Mediterranean *Dactylorhiza* Neck. ex Nevski (Orchidaceae). Causes for taxonomic deficiencies in certain groups may be seen in complex evolution patterns including recent diversification, polyploidy, and/or hybridization related to glaciation events (Abbott et al., 2018; Carnicero et al., 2017; Feliner, 2014; Fiz‐Palacios & Valcárcel, 2013).

Using DNA sequence variation, biogeographic patterns of Mediterranean plants have been analyzed in several studies. An overall general pattern is not yet found and is rather unlikely to exist at all. Feliner (2014) recognized gradual range expansion, vicariance, long‐distance dispersal, radiations, hybridization and introgression, changes in reproductive systems, and colonization abilities as main evolutionary processes in the Mediterranean area. He found varying patterns for different groups of organisms. One of the discussed modes is the direction of migration or dispersal. For example, an eastward expansion from the western Mediterranean was hypothesized for the genus *Narcissus* L. (Amaryllidaceae; Santos‐Gally et al., 2012). In the *Euphorbia myrsinites* L. group (Euphorbiaceae), an amphi‐Tyrrhenian pattern was explained by colonization of the Apennine Peninsula from the Balkan facilitated by Pleistocene land bridges (Falch et al., 2019). Another aspect is the potential for range expansion. Some Mediterranean taxa like the widespread *Narcissus tazetta* L. group show high dispersal and colonization potentials in the entire area (Santos‐Gally et al., 2012). Other taxa like *Abies nebrodensis* (Lojac.) Mattei (Pinaceae; Parducci et al., 2001), a local endemic in Sicily, are restricted to smaller areas.

Across the entire Mediterranean area, but especially in mountainous, species‐rich regions of the Iberian, Apennine, and Balkan Peninsulas, several glacial refugia have been identified for plants and animals (Feliner, 2014; Hewitt, 1999, 2011; Médail & Diadema, 2009; Schmitt, 2007). The spatial and temporal dimensions of persisting series of Quaternary climate fluctuations show a high degree of complexity which may generate different biogeographic histories (Feliner, 2014; Gómez & Lunt, 2007). An example for a group with multiple refugial areas is the *Euphorbia verrucosa* L. alliance (Euphorbiaceae) which survived the ice ages in the Iberian, Apennine and Balkan Peninsulas (Cresti et al., 2019).

**FIGURE 1:**
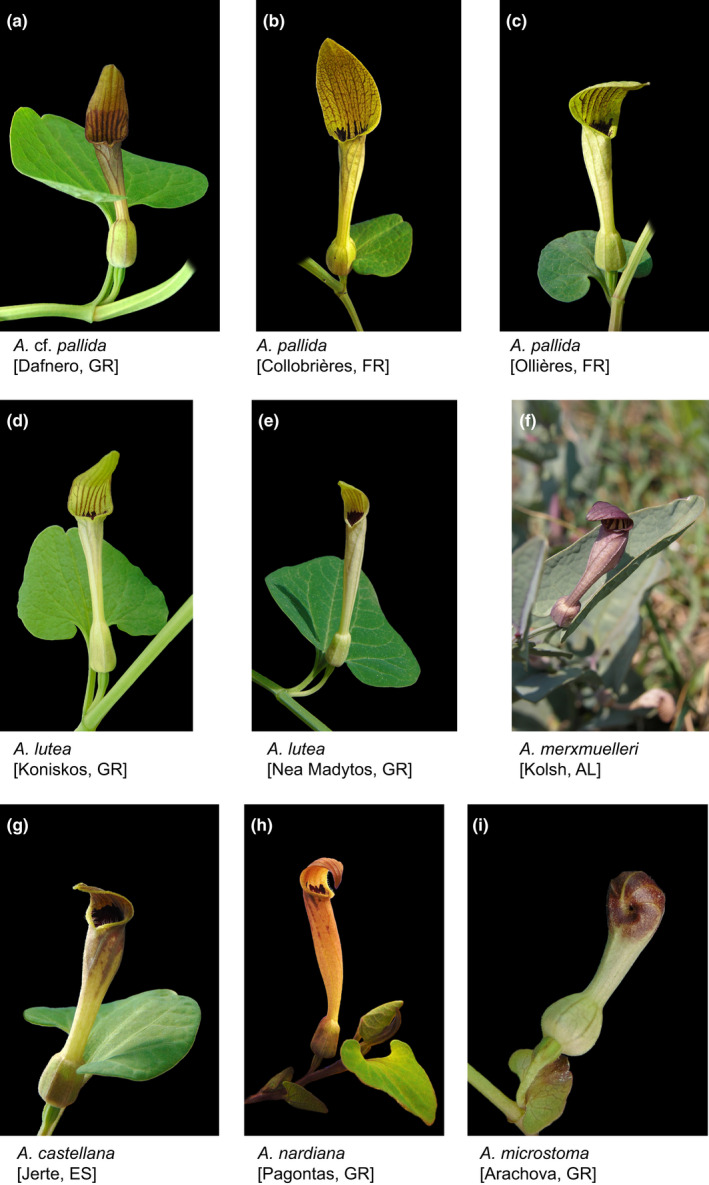
Illustrations of some *Aristolochia* species included in this study. – a–c: *A*. *pallida* (a: Dafnero, Greece; b: Collobrières, France; c: Ollières, France); d–e: *A*. *lutea* (d: Koniskos, Greece; e: Nea Madytos, Greece); f: *A*. *merxmuelleri* from Albania; g: *A*. *castellana* from Spain; h: *A*. *nardiana* from Greece; i: *A*. *microstoma* from Greece. Pictures a‐e and g‐i provided by Dominik Frank, f by Lulezim Shukavon

*Aristolochia* (Aristolochiaceae) is represented with ca. 50 species across the entire Mediterranean basin. For *Aristolochia baetica* L. and *A*. *sempervirens* L., a split between Western Mediterranean populations and Central/Eastern Mediterranean with Southern Moroccan populations has been proposed by Mahfoud (2010). Another subgroup reflecting a pan‐Mediterranean distribution is the *A*. *pallida* Willd. complex. The following taxa have been attributed to this group: *A*. *attica* Orphan. ex Duch., *A*. *attica* Boiss. ex Lojac., *A*. *castellana* (Nardi) Costa, *A*. *croatica* Horvatić, *A*. *longa* De Notaris, *A*. *longa* Boiss., *A*. *lutea* Desf., *A*. *lutea* Gaudin, *A*. *nardiana* I.M. Turner (previously named *A*. *elongata* (Duch.) Nardi; Turner, 2015), *A*. *macedonica* Bornm., *A*. *melanoglossa* Bornm., *A*. *merxmuelleri* Greuter & E. Mayer, *A*. *microstoma* Boiss. & Spruner, *A*. *pallida* Willd., *A*. *sicula* Tineo, and *A*. *tyrrhena* Nardi & Arrigoni (Ball, 1964; Costa, 2008; Horvatić, 1933; Mayer & Greuter, 1985; Nardi, 1984, 1988, 1989, 1991; Nardi & Nardi, 1987; Turner, 2015; Wanke, 2006). The taxonomy of this complex has been the subject of a number of investigations (Nardi, 1984, 1988, 1989, 1991; Nardi & Nardi, 1987; Shuka & Malo, 2011; Trinajstić, 1990; Wanke, 2006). The broad morphological variability (Figure 1) within the commonly accepted species often caused difficulties to assign some specimens to a certain taxon. Meanwhile, many of these taxa have been excluded from the *A*. *pallida* group or were treated as synonyms. Recently, of those species, only *A*. *pallida*, *A*. *nardiana* (as *A*. *elongata*), *A*. *microstoma*, and *A*. *merxmuelleri* were recognized by Wanke (2006). He distinguished *A*. *microstoma* and *A*. *nardiana* from *A*. *merxmuelleri* and *A*. *pallida* by sharing an elongated rootstock and *A*. *microstoma* by its unique perianth morphology. *Aristolochia pallida* and *A*. *merxmuelleri* are characterized by a globose tuberous tuber.

First phylogenetic analyses by Wanke (2006) found *A*. *pallida* and *A*. *lutea* intermingled in one clade, as sister to *A*. *merxmuelleri*. Accordingly, it was advised to sink *A*. *lutea* into *A*. *pallida*. The weakly supported sister clade to this group comprised *A*. *microstoma* and *A*. *nardiana*. In Wanke (2006) *A*. *pallida* and *A*. *lutea*, occurring from France to Turkey, were sampled with a very limited number of accessions. We here extent phylogenetic analyses with a comprehensive sampling of the *A*. *pallida* group covering its entire distribution range to address the following questions.

1. Are the recognized taxa in the *A*. *pallida* complex monophyletic?
2. Can lineages in the *A*. *pallida* group be interpreted as glacial relicts based on age estimations?
3. Do they correspond to geographic units on the regional scale and/or in terms of hypothesized glacial refugia in the Mediterranean?

## 2 MATERIALS AND METHODS

### 2.1 Taxon sampling

Based on Wanke (2006) and Costa (2008), we included all currently accepted species of the *A*. *pallida* complex in our analysis (*A*. *pallida*, *A*. *lutea*, *A*. *nardiana*, *A*. *microstoma*, *A*. *merxmuelleri*, *A*. *croatica*, and *A*. *castellana)*. In total, 87 accessions of the aforementioned taxa covering both the entire distribution range as well as the phenotypic diversity were included. The phenotypic diversity of our ingroup taxa is illustrated in Figure 1. The distribution of the *A*. *pallida* complex as well as the origin of the sampled accessions is shown in Appendix S1. Additional 41 accessions of 33 *Aristolochia* taxa were selected to include the entire diversity of *Aristolochia* section *Diplolobus* (subsection *Aristolochia* and *Podanthemum*) as well as more distantly related outgroups from the remaining *Aristolochia* lineages (Wanke, 2006; Wanke et al., 2006, 2007). The Mediterranean *Aristolochia* species belong to *Aristolochia* subsection *Aristolochia*, whereas *Aristolochia* subsection *Podanthemum* is distributed from Asia to Africa. Both taxa belong to *Aristolochia* subgenus *Aristolochia*. *Aristolochia* subgenus *Siphisia* is sampled with three accessions (*A*. *westlandii*, *A*. *salvadorensis*, and *A*. *macrophylla*). The material is listed in Appendix S1.

**TABLE 1:** Overview of the two clades with ten major groups (see also Figures 2 and 3, numbers in boxes) revealed by ML, BI, and haplotype network analyses

Group number	Clade	Taxonomy	Geography	PP value	BS value
1	Greek	*A. nardiana*	Peloponnese, central Greece and an accession from Thessaly	1	97
2		*A*. *microstoma* and one accession of *A*. *nardiana*	eastern Peloponnese and eastern central Greece and an accession from Euboea	1	98
3		*A*. *nardiana*, *A*. *lutea* and *A*. cf. *pallida*	Macedonia (Greece) and Thessaly	1	81
4	Pan‐Mediterranean	*A. merxmuelleri*	Albania and Kosovo	1	100
5		*A*. *pallida*, including *A*. cf. *lutea*	North Western Turkey	1	88
6		*A. castellana*	Spain	0.99	64
7		*A*. *castellana* and *A*. *pallida*	Spain and France		
8		*A*. *lutea* and *A*. *pallida*	Italy		
9		*A*. *lutea*, *A*. *pallida* and *A*. *croatica*	Croatia and Italy		
10		*A. lutea*	Slovenia, Croatia, and Italy		

### 2.2 DNA extraction, PCR amplification and sequencing

Total genomic DNA was extracted from herbarium specimens, fresh leaves of cultivated plants, and silica dried leaf material, collected in natural populations, respectively. DNA extraction was performed using a double‐extraction approach with CTAB according to Borsch et al. (2003) or employing the extraction kit NucleoSpin Plant II (Macherey‐Nagel, Düren, Germany) following the manufacturer's protocol. Several DNA markers were tested for the selected accessions. The amplification of nrITS using the standard primers ITS‐A, ITS‐B, ITS‐C, and ITS‐D (Blattner, 1999) was not successful. Consulting Mahfoud (2010), who used a nuclear single copy gene of the S8e family (ribosome biogenesis) for the *A*. *sempervirens* complex, we also evaluated this marker. It yielded, however, no variation for our ingroup. Referring to Wanke et al. (2006); Wanke et al. (2007), we used the chloroplast *trnK*‐*matK*‐*psbA* region for phylogenetic reconstructions (i.e., *trnK* intron, *matK* gene, and *trnK*‐*psbA* spacer).

All PCRs were performed with a Sensoquest or Biometra cycler under the following conditions: 5 min 94°C, 30 cycles (30 s 94°C, 30–45 s *T_a_*, 90–180 s 72°C), 10 min 72°C. Touch down PCR profiles included 10 initial cycles starting at 5°C above the later used annealing temperature *T_a_*, which was 5°C below the melting temperatures of the used primers. The reaction mixture consisted of 1× PCR reaction buffer (containing 2 mM MgCl_2_), 0.2 mM dNTPs, 0.4 µM each of forward and reverse primer, 0.05 U/µl DreamTaq DNA polymerase (Thermo Fisher Scientific), and 1 µl of the DNA extract as template. Amplification was done in a single fragment employing the primers *trn*K‐F (Wicke & Quandt, 2009) and *psb*A‐R (Steele & Vilgalys, 1994). If this was not successful the region was divided in multiple smaller and overlapping fragments employing following primers: trnK‐3914F (Johnson & Soltis, 1994), AR‐matK‐1200F, AR‐matK‐1510R, AR‐matK‐2510R, AR‐matK‐2100R, AR‐matK‐2400R (Wanke, 2006), and Ari‐trnK‐1938F: TGGCAGTGTTATTTTCACTTGTGG, Ari‐trnK‐2466F: TCCAAGAACCTCTTCTATTTCGCA, Ari‐trnK‐2756R: TTGCACACGGCTTTCCCTAT, Ari‐trnK‐3077R: TGGAGGGCTTGTTATTCAACAGT (all this study).

PCR products were cleaned with the NucleoSpin Gel and PCR Clean‐up kit (Macherey‐Nagel, Düren, Germany). If necessary, PCR products were purified using a 1.2% agarose extraction gel and the NucleoSpin Extract II‐Kit (Macherey‐Nagel). Both strands were obtained for each PCR product using the PCR primers and BigDye Terminator v3.1 Cycle Sequencing Kit (Thermo Fisher Scientific, Carlsbad, CA, USA). External sequencing service was provided by LGC Genomics, Berlin and by Macrogen Europe, Amsterdam. Sequence files were either checked and consensus sequences were compiled using GENEIOUS version 11.1.5 (http://www.geneious.com, Kearse et al., 2012) or manually edited employing the Phylogenetic Data Editor PhyDE v.0.995 (www.phyde.de). All newly generated sequences were deposited at GenBank of the National Center for Biotechnology Information (NCBI), the corresponding accession numbers are listed in Appendix S1.

### 2.3 Phylogenetic analyses

Sequences were aligned using MAFFT v7.388 (Katoh et al., 2002) as implemented in GENEIOUS followed by a manual check according to the alignment rules proposed by Kelchner (2000) and Borsch et al. (2003). Some very variable poly A‐T regions were excluded from the analysis. This concerns the alignment positions 1–64, 377–1143, 1243–1257, 1471–1480, 1532–1566, 2341–2358, 3363–3387, 3695–3709, 3857–3955, and 4009–4061. The alignment is deposited in Dryad (https://doi.org/10.5061/dryad.n5tb2rbxp). Bayesian Inference (BI) and Maximum likelihood (ML) trees were calculated using MrBayes (Huelsenbeck & Ronquist, 2001) and PHYML (Guindon & Gascuel, 2003) in GENEIOUS. According to the outcomes of JMODELTEST (Darriba et al., 2012), we used the GTR model with four Gamma categories, with estimated proportion of variable sites and gamma distribution parameters. For MrBayes, the chain length was 1,100,000 generations, four heated chains with temperature of 0.2, a sample frequency of 200, and a burn‐in of 10%. Convergence of chains and effective sample size (ESS) values for the independent runs were evaluated within GENEIOUS. For ML, nearest‐neighbor interchange was used for the tree searches. Using the same options, 1000 ML bootstrap (BS) replicates were conducted.

### 2.4 Divergence time estimation

To evaluate divergence times for the *Aristolochia pallida* complex, we used Bayesian inference (BI) implemented in BEAST v2.5 (Drummond et al., 2012). As calibration we used fossils described from the late Miocene in Austria (Meller, 2014). These leaf impressions, described as *Aristolochia austriaca* Meller, are the most reliable paleontological records of the genus among the few *Aristolochia* fossils (Meller, 2014). They were found in the Hollabrunn‐Mistelbach Formation which is dated to 11.0 or 11.1 Ma (Harzhauser et al., 2011; Roetzel et al., 1999). The author discussed similarities of these fossils and the extant *A*. *rotunda* and *A*. *baetica*. We followed this interpretation and used these dates in a first step dating analyses of *Aristolochia* species with a broad selection of European *Aristolochia* species and a reduced taxon set of the *Aristolochia pallida* group (= Beast 1 analysis). For this analysis, *A*. *salvadorensis*, *A*. *westlandii*, and *A*. *macrophylla* were used as the outgroup. We modeled the most recent common ancestor (mrca) of *A*. *rotunda* and *A*. *baetica* 11.0 Ma in a normal distribution with sigma set to 0.1. This normal distribution reflects the small interval of 11.0–11.1 Ma in the geological age estimation of the Hollabrunn‐Mistelbach Formation. We used a relaxed lognormal clock, the Yule model, and the GTR substitution model with four Gamma categories and the shape being estimated. This result was used to date evolutionary splits within the *Aristolochia pallida* complex (= Beast 2 analysis). Here, we used the coalescence constant population model because of the inclusion of a large number of intraspecific accessions. We also applied the GTR substitution model. The mrca of the *Aristolochia pallida* group was set to an age of 1.9867–4.7541 Ma (see results) in a uniform prior. Runs of 20,000,000 generations with samples taken every 2000 generations provided ESS values >200 for all analyses in TRACER (Rambaut & Drummond, 2007). Due to low support values in several clades, we refrained from applying biogeographic ancestral area analyses.

### 2.5 Haplotype network

The cpDNA dataset was analyzed through a statistical parsimony algorithm (Templeton et al., 1992), as implemented in TCS 1.21 (Clement et al., 2000), to infer genealogical relationships among haplotypes. The maximum number of differences resulting from single substitutions among haplotypes was calculated with 95% connection limit. Gaps were treated as missing data. The network was edited using tcsBU (Múrias dos Santos et al., 2016).

## 3 RESULTS

### 3.1 Phylogenetic analyses and Haplotype network

**FIGURE 2:**
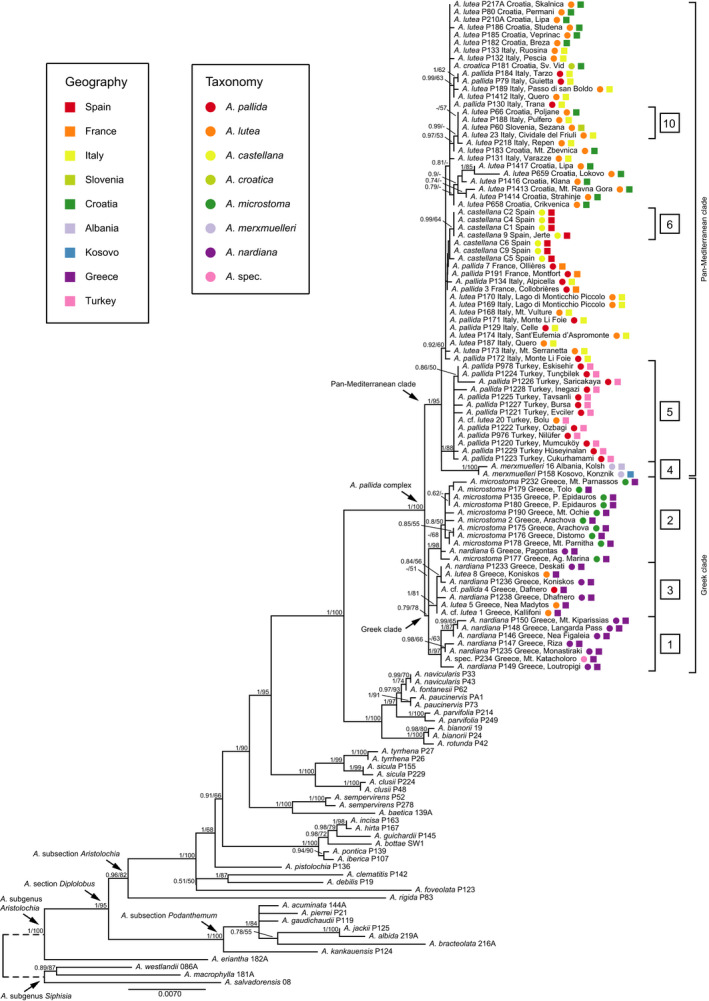
Phylogenetic Maximum likelihood tree based on *trnK*‐*matK* sequences of *Aristolochia* species with focus on the *A*. *pallida* group. Numbers at branches indicate PP and BS (≥50%) values. Dotted line marks branch length that was longer in the analysis. Sample numbers and (original) geographical provenances are given after the taxon names. Taxa and geographical provenances are coded by color. Brackets with numbers in boxes represent major groups of the *A*. *pallida* complex

Our study generated 99 new sequences of the *trnK*‐*matK*‐*psbA region*. The aligned sequence length was 4061 bp. The BI analyses showed rapidly converging chains and yielded trees with a mean log‐likelihood of −9645.648 with a standard deviation of 0.928, highest posterior density (HPD) ranges from −9672.864 to −9620.74 and an ESS of >200. The ML tree (Figure 2) had a log‐likelihood of −9470.71632. Pairwise distances varied between 0 and 0.0106 in the ingroup and 0 and 0.0958 in the entire alignment. There were no topological differences between the BI and ML analyses.

*Aristolochia* subsection *Podanthemum* is recovered monophyletic (1/100) with *A*. *kankauensis*, *A*. *bracteolata*, *A*. *albida*, *A*. *jackii*, *A*. *gaudichaudii*, *A*. *pierrei*, and *A*. *acuminata*.

A grade is recovered for *Aristolochia* subsection *Aristolochia* with *A*. *rigida* (endemic to Somalia) branching first, followed by temperate Asian and temperate Eurasian representatives (i.e., *A*. *foveolata*, *A*. *debilis*, *A*. *clematitis*) and *A*. *pistolochia*. The latter is endemic to Southern France and Spain. The following sister groups consist of 1) the Caucasian, Near East, and East Mediterranean species *A*. *iberica*, *A*. *pontica*, *A*. *bottae*, *A*. *guichardii*, *A*. *hirta*, and *A*. *incisa* (1/100), and 2) the core West Mediterranean species. The latter include *A*. *sempervirens* and *A*. *baetica* in a clade (1/100), followed by a clade (1/100) with *A*. *sicula* (endemic to Sicily), *A*. *thyrrena* (endemic to Sardinia and Corsica), and *A*. *clusii* (endemic to southern Italy). Furthermore *Aristolochia navicularis (Italy*, *Tunesia)*, *A*. *fontanesii (Algeria)*, *A*. *paucinervis (France*, *Spain*, *Morocco)*, *A*. *parvifolia (Greece)*, *A*. *bianorii* (endemic to the Balearic Islands), and *A*. *rotunda* (widespread in the Western Mediterranean) are sister groups (1/100) to the *A*. *pallida* complex.

The ML and BI analyses resulted in two clades within the *A*. *pallida group* (Figure 2). One is rather poorly supported (PP 0.79/BS 78, but 0.99 PP in the Beast 2 analysis, Figure 6) and comprises Greek accessions of *A*. *nardiana*, *A*. *microstoma*, *A*. *lutea*, and *A*. *pallida*. It is referred to as the “Greek group/clade.” The second major clade is better supported (1/95) and includes *A*. *merxmuelleri*, *A*. *croatica*, *A*. *castellana*, *A*. *pallida* from Turkey, France and Italy and *A*. *lutea* from Turkey, Italy, Slovenia, and Croatia. We term it the “pan‐Mediterranean group/clade.” Our ML, BI, and haplotype network analyses revealed the following major groups (Table 1, 2, 3, 4, numbers in boxes).

**FIGURE 3:**
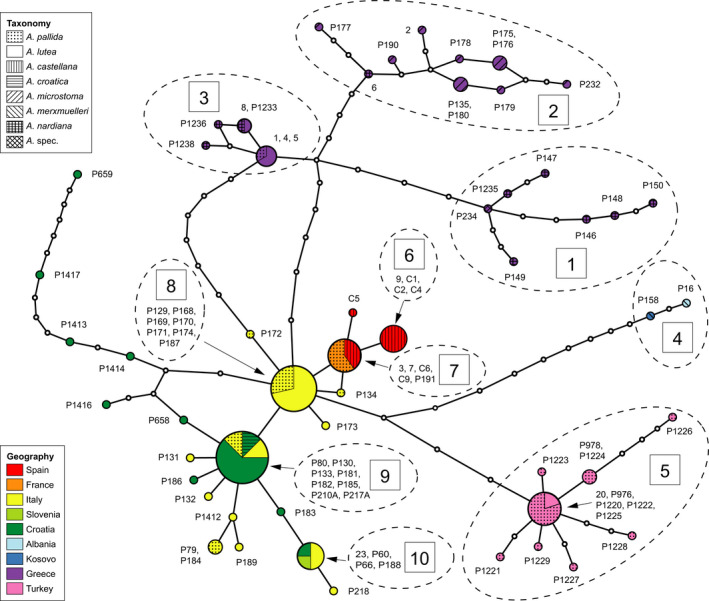
Statistical parsimony network of cpDNA (*trnK*‐*matK*) haplotypes of the *Aristolochia pallida* complex. Circle sizes are proportional to frequencies, lines represent mutational steps, and small white dots are unsampled haplotypes. Numbers in boxes indicate major groups of the *A*. *pallida* complex. Geographical provenances are coded by color, taxa by pattern

**FIGURE 4:**
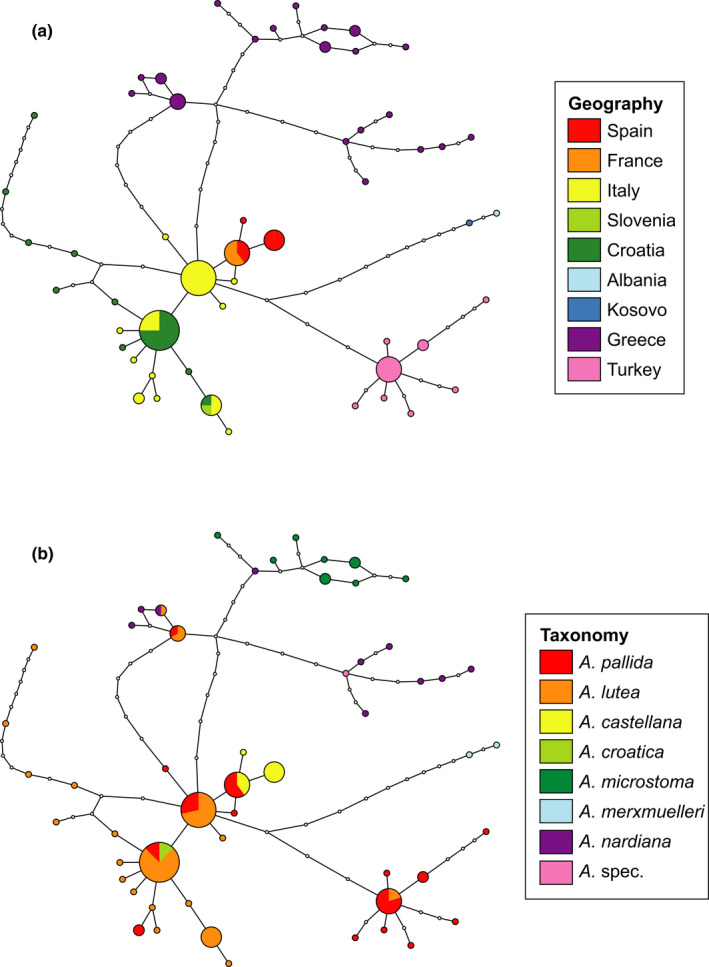
Same haplotype network of *Aristolochia pallida* group as in Figure 3. Circle sizes are proportional to frequencies, lines represent mutational steps, and small white dots are unsampled haplotypes. (a) Geographical provenances are coded by color. (b) Taxa are coded by color

Within the Greek clade:

1. *A*. *nardiana* from Peloponnese and central Greece and an accession from Thessaly (P234) (PP 1, BS 97);
2. Greek *A*. *microstoma* from eastern Peloponnese and eastern central Greece including one accession of *A*. *nardiana* from Euboea (6) (PP 1, BS 98);
3. *A*. *nardiana*, *A*. *lutea*, and *A*. cf. *pallida* from Macedonia (Greece, P1233) and Thessaly (PP 1, BS 81).

Within the pan‐Mediterranean clade:

4. *A*. *merxmuelleri* from Albania and Kosovo (PP 1, BS 100);
5. *A*. *pallida*, including *A*. cf. *lutea* from North Western Turkey (PP 1, BS 88);
6. *A*. *castellana* from Spain (PP 0.99, BS 64);
7. an haplotype consisting of Spanish *A*. *castellana* and *A*. *pallida* from France;
8. an haplotype of Italian *A*. *lutea* and *A*. *pallida*;
9. an haplotype including *A*. *lutea* from Croatia and Italy, *A*. *pallida* from Italy, and *A*. *croatica* from Croatia;
10. an haplotype comprising *A*. *lutea* from Slovenia, Croatia, and Italy.

The remaining haplotypes are mostly unique and dispersed within the pan‐Mediterranean group.

### 3.2 Divergence time estimation

**FIGURE 5:**
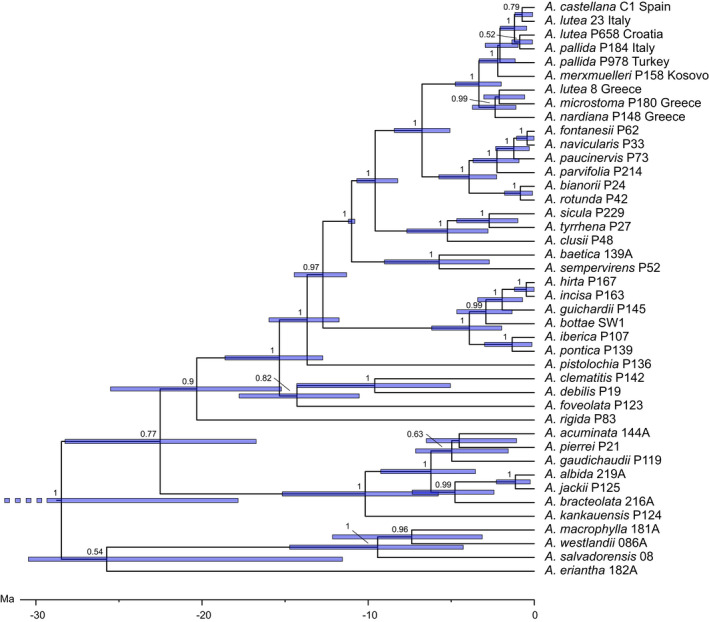
Phylogenetic Maximum Clade Credibility (MCC) tree of the BEAST analyses of *Aristolochia* species with a broad selection of European *Aristolochia* species and a reduced taxon set of the *Aristolochia pallida* group (= Beast 1 analysis). Bars at the nodes indicate 95% highest posterior densities; posterior probabilities ≥ .50 are given above the branches

**FIGURE 6:**
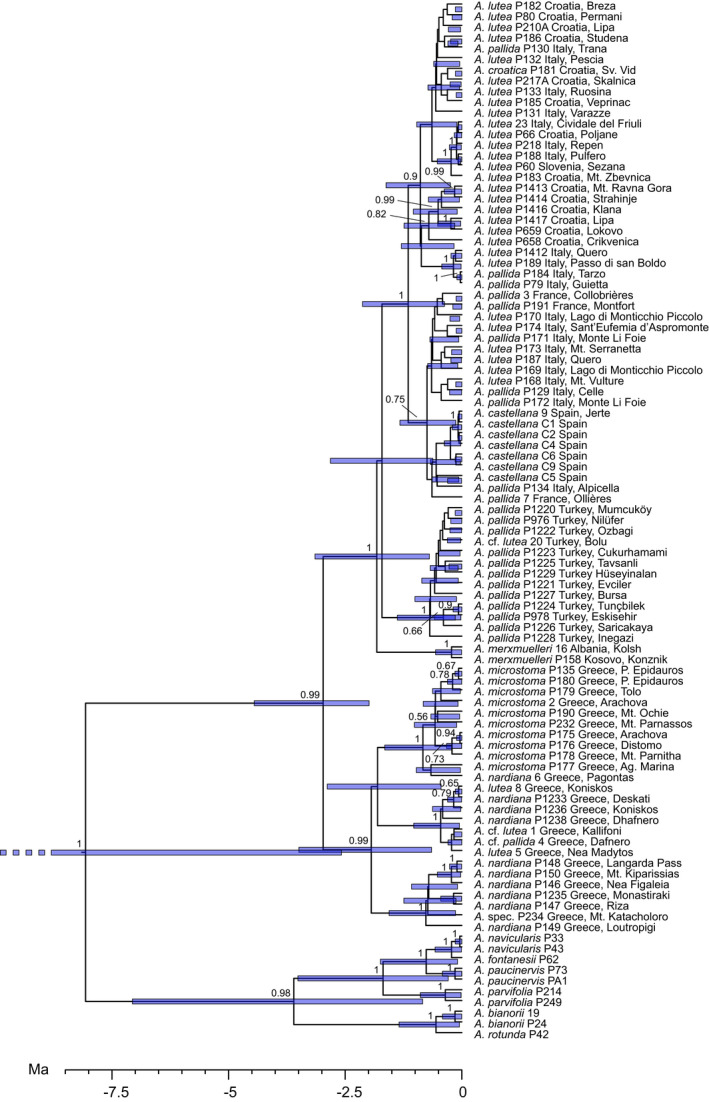
Phylogenetic Maximum Clade Credibility (MCC) tree of the BEAST analyses of evolutionary splits within the *Aristolochia pallida* complex (= Beast 2 analysis). Bars at the nodes indicate 95% highest posterior densities; posterior probabilities ≥ .50 are given above the branches

The Beast 1 analysis (Figure 5) focusing on interspecific relationships within *Aristolochia* dated the crown node of the *Aristolochia pallida* group to 3.33 Ma (mean), 3.27 Ma (median), 1.99–4.75 Ma (95%HPD). Moreover, the following crown node ages were revealed: *Aristolochia* subsection *Aristolochia* (incl. *A*. *rigida*) 20.32 Ma (mean), 19.59 Ma (median), 15.22–25.51 Ma (95%HPD), *Aristolochia* subsection *Podanthemum* 10.17 Ma (mean), 9.91 Ma (median), 5.78–15.17 Ma (95%HPD), *Aristolochia* subgenus *Siphisia* 9.43 Ma (mean), 9.13 Ma (median), 4.27–14.72 Ma (95%HPD). According to the Beast 2 analysis (Figure 6), the mrca of the Greek clade is 1.93 Ma (mean), 1.83 Ma (median), 0.65–3.49 Ma (95%HPD) and the mrca of the pan‐Mediterranean clade is 1.82 Ma (mean), 1.72 Ma (median), 0.69–3.15 Ma (95%HPD) old.

## 4 DISCUSSION

### 4.1 Taxonomy

Our phylogenetic reconstruction of the *A*. *pallida* complex is based on the plastome *trnk*‐*matK*‐*psbA* region which has previously been suggested to provide good support and resolution on species level (Shaw et al., 2005). In our group, this marker shows low variation rates within a species complex but rather good resolution on species level in *Aristolochia* (Figure 2). Despite the limitation within the *A*. *pallida* complex, our molecular data allow conclusions on the taxonomy of the *A*. *pallida* group. Accordingly, most of the current taxonomic treatments are not corroborated. In particular, *A*. *pallida* and *A*. *lutea* are not only placed in different clades, especially in the pan‐Mediterranean clade, but also to a lesser extent in the Greek clade. Traditionally, *A*. *pallida* and *A*. *lutea* have been distinguished by limb‐tube ratio, <1 for *A*. *lutea* and ≥1 for *A*. *pallida*, and by chromosome numbers (*A*. *lutea* 2*n* = 8, *A*. *pallida* 2*n* = 10; Nardi, 1984). These morphotypes are dispersed throughout the phylogenetic tree and the haplotype network. The phylogenetic relationships argue for a high morphological variation within geographic subclades (Figure 4). The diverse flower morphology may be linked to the specialized pollination syndromes in this group (Rulik et al., 2008). Comparable results were found for the Turkish *A*. *hirta* group, indicating that morphological variation could have been conserved in refugial areas or evolved recently, also as result of hybridization (Mahfoud, 2010). Despite slight morphological differences, *A*. *lutea*, described in 1808, should be sunk into the earlier, in 1805 described *Aristolochia pallida*, confirming Wanke (2006). Because *A*. *croatica* shares the same haplotypes (group 9, Figure 3) as *A*. *lutea* and *A*. *pallida* the maintenance as its own species is not warranted.

Costa (2008) raised *A*. *pallida* ssp. *castellana* into species rank. The morphological characters including tuber shape and chromosome numbers of 2n=10 of *A*. *castellana* fall into the range of *A*. *pallida* s.l. *Aristolochia castellana* is represented in three haplotypes, one of which (group 7, Figure 3) also includes *A*. *pallida* from France. Therefore, its treatment as own species may be questioned.

*Aristolochia merxmuelleri* has been described on the basis of its morphological characters, color, and distribution (Mayer & Greuter, 1985; Shuka & Malo, 2011). It differs from other taxa of the *A*. *pallida* from the Balkan by the diminutive size, triangular to almost sagittate‐reniform leaves, a pronounced hump on the back, the length of the flower peduncles, and an ovary which is longer than the petiole in fully developed plants. Indeed, the distinctiveness of *A*. *merxmuelleri* is supported also by our molecular data possessing own haplotypes and appearing a single clade (group 4) in the phylogenetic trees (Figures 2 and 3, Table 1). Previously, *A*. *merxmuelleri* was only known from serpentine areas in Kosovo. Recent reports (Shuka et al., 2011) indicate that *A*. *merxmuelleri* is present in northeast Albania. In its distribution range, *A*. *merxmuelleri* overlaps with other species of the *A*. *pallida* complex. Therefore, a denser sampling in the future might breakup the monophylly of *A*. *merxmuelleri* as well.

Morphologically, the Greek *A*. *microstoma* can be clearly separated from all other species by unique fyke‐shaped perianth with a very narrow entrance, flowers usually appear at ground level in the leaf‐litter or between rocks (Rupp et al., 2021; Wanke, 2006). Our molecular data mainly confirm to treat *A*. *microstoma* as an evolutionary unit (group 2). Only one accession morphologically identified as *A*. *nardiana* (6), but occurring close to the area of distribution of *A*. *microstoma*, namely Euboea, is found inside the clade of *A*. *microstoma* (Nardi, 1991). Recently the pollination biology of *A*. *microstoma* was studied. Pollinator deception is likely mediated by chemical components typically released from dead insects, postulating that pollinators are likely deceived by chemical imitation of invertebrate carrion, a deceptive strategy not described from another plant species so far (Rupp et al., 2021). Further studies are needed to confirm if the abovementioned accession identified as *A*. *nardiana*, and thus morphologically very different from *A*. *microstoma*, which has a similar floral scent to *A*. *microstoma*, and thus employed similar pollinating fly species.

*Aristolochia nardiana* has been delimited from *A*. *pallida* and *A*. *lutea* by its perianth shape and an elongated tuber (Nardi, 1989). Although the aerial characteristics within *A*. *nardiana* seem to be stable, numerous *A*. *lutea* morphotypes from all over the distribution area of *A*. *pallida* s.l. show similar features. The tuber shape, long versus globose, as diagnostical character is relativized by the infraspecific variation in *A*. *nardiana* ranging from ellipsoid to slender shapes (Nardi, 1989). This shape may be linked to ecological parameters, for example, varying precipitation quantities in combination with different substrates. Similar observations have been found for *A*. *rotunda* s.l. where globose and elongate (subsp. *insularis*) tubers occur throughout the southern distribution range (Nardi, 1991). A better trait to characterize Peloponnese and adjacent populations of *A*. *nardiana* are presumably hirsute perianth surfaces. Overall, the morphological discrepancies between *A*. *nardiana* and *A*. *pallida* s.l. are, however, rather minor. In our analysis, *A*. *nardiana* is part of the Greek clade (Figure 2, Table 1), it falls, however, into a core *A*. *nardiana* group from Peloponnese and central Greece (group 1) and a heterogeneous group with accessions of *A*. *lutea* and *A*. *pallida* from Macedonia and Thessaly In Greece (group 3). Therefore, the species delimitation may be questionable.

Applying a strict phylogenetic species concept to the *A*. *pallida* complex would imply either to lump all taxa into one *A*. *pallida* or to describe a series of new species or subspecies to retain monophyly. However, new species or subspecies would be very difficult to identify morphologically and would thus not help, for example, local floristic treatments and thus the general acceptance. We here refrain from formally drawing taxonomic conclusions given that until now only plastome‐derived molecular markers were used and those results will need confirmation by nuclear‐derived loci either substantiating our findings or providing an alternative evolutionary scenario.

### 4.2 Biogeography

The evolutionary patterns of the *A*. *pallida* complex deviate from former taxonomic concepts (2, 3, 4). It opens the question whether the taxa may represent glacial relicts, an aspect that may have been disregarded when establishing morphology‐based taxonomy. Based on our molecular clock analyses, the crown node of the *A*. *pallida* complex is dated to the Upper Pliocene, being about 3.0–3.3 Ma old, before the onset of glaciation cycles in the Pleistocene. The differentiation processes within the Greek and the pan‐Mediterranean clades, however, fall into European glaciation times. Therefore, the temporal patterns support an interpretation that the evolutionary patterns were highly influenced by Pleistocene climate changes.

Our data contain a strong geographic signal (Figure 4), especially in longitudinal direction, which is a frequent pattern in Mediterranean taxa (Feliner, 2014). The Greek clade is exclusively distributed in Greece and haplotype networks including the sister group of the *A*. *pallida* complex identify this region as a potential source area for the entire group. Within the Greek clade (Figures 2 and 3, Table 1), a certain substructure is visible, the *A*. *nardiana* group (group 1) in SW‐W Greece, *A*. *microstoma* (group 2) in eastern Greece, and the mixed *A*. *nardiana*, *A*. *lutea*, and *A*. cf. *pallida* (group 3) in central and northeastern Greece. This may hint toward different glacial refugia areas on the Aegean microplate from which diversification and range extension started. This pattern may be congruent with the “refugia within refugia” hypothesis by Gómez and Lunt (2007), originally developed for the Iberian peninsula. An East Mediterranean, for example, Anatolian, origin was also found for the stem node of *Echium*, *Borago*, and *Anchusa* s.l. clades of Boraginaceae by Mansion et al. (2009). Still, as Feliner (2014) pointed out, patterns of glacial refugia and range expansion in the Mediterranean are often very complex and different spatial and temporal levels need to be distinguished. Using the present data, we restrict conclusions concerning the evolutionary history of the *A*. *pallida* group to larger geographical scales.

The pan‐Mediterranean clade contains central and western Mediterranean and North Western Turkish groups. Within the *A*. *pallida* complex, *A*. *pallida* and *A*. *lutea* disintegrate into several supported geographic subclades. These subclades may likely represent regional radiations out of different glacial refugia in the Mediterranean. The Italian peninsula represented by *A*. *lutea* and *A*. *pallida* (group 8, Figure 3) occupies a central position in the haplotype network. This haplotype is closely related to several amphi‐Adriatic groups mainly containing not only *A*. *lutea* but also *A*. *pallida* and *A*. *croatica*. Similar biogeographic patterns have been found for the amphi‐Adriatic *Campanula garganica* Ten. clade (Campanulaceae; Park et al., 2006) and for *Euphorbia myrsinites* L. (Euphorbiaceae; Falch et al., 2019). Park et al. (2006) roughly estimated the origin of the disjunct *Campanula* lineage to the early to late Pleistocene. This corresponds well to our molecular clock analyses dating these events also to the late Pleistocene. Northern Istria is well represented by *A*. *lutea* and *A*. *croatica* in our datasets. It is one of several hypothesized refugia areas for the Western Balkan (Médail & Diadema, 2009). For *Salvia officinalis* L. (Lamiaceae), Jug‐Dujaković et al. (2020) hypothesized two glacial refugial areas in the Western Balkans and subsequent range extension. Northern Istria may thus have served as a refugia from which the Italian peninsula may have been repeatedly colonized, possibly facilitated by land bridge connections for direct migration routes during Pleistocene sea level fluctuations.

*Aristolochia merxmuelleri* (group 4, Figures 2 and 3) is clearly separated from other taxa, possibly since the early Pleistocene (Figure 6). This may argue to interpret its limited present area of distribution in Kosovo and northeast Albania (Shuka et al., 2011) as a long‐term glacial refugium. A range extension of this stenoecious taxon may have been hampered by its specific ecological preference to a serpentine rocky substratum which is geographically restricted to few areas in the Western Balkans (Mayer & Greuter, 1985).

The westernmost distributed taxon is *A*. *castellana* with a narrow distribution in Central Spain (Costa, 2008). Costa (2008) proposed that *A*. *castellana* is a relict, paleoendemic taxon. This would imply that Iberia may have served as source area for a postglacial range extension in eastwards direction. In our analyses, this species has two exclusive haplotypes and one which is shared with southern French *A*. *pallida* (group 7, Figure 3). These haplotypes are closely related to Northern Italian *A*. *pallida* and *A*. *lutea* (group 8, Figure 3). Thus, our data reject the hypothesis that *A*. *castellana* is a paleoendemic relict. First, it is dated to the late Pleistocene and second, it has a derived position in the haplotype network. This rather favors to interpret it as a result of relatively recent dispersal or late Pleistocene migration from a Southern French and Northern Italian refuge to the Iberian peninsula (cf. Feliner, 2014).

The clade of northeastern Turkish *A*. *pallida* (group 5, Figures 2 and 3) is molecularly well distinguished from other groups and may have originated in the early Pleistocene, around 1.6/1.7 Ma (Figure 6). It likely represents a lineage which survived glaciation periods in this region. Its area of distribution falls outside the main centers of endemism in Turkey (Noroozi et al., 2019), however, it flanks a species‐rich region in the west of the North Anatolian Mount chain and north of the West Anatolian Taurus. Turkish *A*. *pallida* exemplifies a high potential for Pleistocene range extension.

Médail and Diadema (2009) proposed about 50 glacial refugia in the Mediterranean. Compared to the distribution of our groups and subclades they are spatially limited to smaller areas. Of those, northern Istria, maritime Alps, Alpi Apuani, and Peloponnese match accessions sampled in our study. Answering the question whether the generally hypothesized glacial refugia can be attributed to the *A*. *pallida* group would require an even denser taxon sampling. Because these endemic rich areas are often found in Mediterranean mountains (Médail & Diadema, 2009; Noroozi et al., 2019) and members of the *A*. *pallida* complex are not restricted to high altitudes, survival during ice ages outside these classic areas, but in vicinity to them, seems possible. Hughes et al. (2006) found high dynamics in the Late Pleistocene glaciers in the Pindus Mountains in Greece leading to a variety of local climates, including potentially suitable ones for *Aristolochia* taxa, depending on altitudinal range in this mountain region.

Our data illustrate that lineages dated back to the early Pleistocene like *A*. *merxmuelleri* are restricted to a very limited area of distribution, while others like the Turkish *A*. *pallida* clade or *A*. *castellana*/French *A*. *pallida* exemplify a relatively high potential for range extension. To conclude, the *A*. *pallida* group shows a diverse biogeographic pattern of different glacial refugia throughout the Mediterranean region (Feliner, 2014), including some subclades with limited dispersal capabilities at the regional scale and others with much wider range extension and some disjunctions where long distance dispersal may have been involved.

## CONFLICT OF INTEREST

The authors declare no conflict of interest.

## AUTHOR CONTRIBUTIONS

**Cornelia Krause:** Formal analysis (equal); Investigation (equal); Visualization (equal); Writing – original draft (equal); Writing – review & editing (equal). **Birgit Oelschlägel:** Formal analysis (equal); Investigation (equal); Resources (equal); Writing – review & editing (equal). **Hafez Mahfoud:** Investigation (equal); Resources (equal); Writing – review & editing (supporting). **Dominik Frank:** Investigation (equal); Resources (equal); Writing – review & editing (supporting). **Gérard Lecocq:** Resources (equal); Writing – review & editing (supporting). **Lulëzim Shuka:** Resources (equal); Writing – review & editing (supporting). **Christoph Neinhuis:** Resources (equal); Writing – review & editing (supporting). **Pablo Vargas:** Investigation (equal); Writing – review & editing (supporting). **Aycan Tosunoglu:** Resources (equal); Writing – review & editing (supporting). **Mike Thiv:** Conceptualization (equal); Formal analysis (equal); Investigation (equal); Writing – original draft (equal); Writing – review & editing (equal). **Stefan Wanke:** Conceptualization (equal); Formal analysis (equal); Investigation (equal); Resources (equal); Writing – review & editing (equal).

## Supporting information

Appendix S1

## Data Availability

Data associated with this manuscript are stored in the Dryad Digital Repository (https://doi.org/10.5061/dryad.n5tb2rbxp). Alignment. Nexus file of *Aristolochia* species based on *trnK*‐*matK*.
