# Supplementary material for: The evolution of the Aristolochia pallida complex (Aristolochiaceae) challenges traditional taxonomy and reflects large‐scale glacial refugia in the Mediterranean
Source: Ecol Evol. 2022 Mar 31;12(4):e8765. doi: 10.1002/ece3.8765 (PMC8969917; doi:10.1002/ece3.8765)
Supplement: Supplementary file 1 — Appendix S1 [file ECE3-12-e8765-s001.docx]

**Supporting Information (Appendix)**

Appendix Table 1. Material used for phylogenetic analyses. Taxa with their origin, voucher specimens and GenBank numbers.

| **Taxon** | **Lab No.** | **Origin** | **GPS data** | **Leg. (collection date) & Det.** | **Herbar / Botanic Garden Acc. No.** | **GenBank No. (* this study)** |
| --- | --- | --- | --- | --- | --- | --- |
| *A. castellana* | C1 | Spain |  | Andrea Costa C1 |  | OK019253 |
| *A. castellana* | C2 | Spain |  | Andrea Costa C2 |  | OK019254 |
| *A. castellana* | C4 | Spain |  | Andrea Costa C4 |  | OK019255 |
| *A. castellana* | C5 | Spain |  | Andrea Costa C5 |  | OK019256 |
| *A. castellana* | C6 | Spain |  | Andrea Costa C6 |  | OK019257 |
| *A. castellana* | C9 | Spain |  | Andrea Costa C9 |  | OK019258 |
| *A. castellana* | 9 | Spain |  | Lecocq, G. A12 | STU SMNS-STU-PH-0099629 | MK095589 |
| *A. croatica* | P181 | Croatia, Isle of Pag, Mt. Sveti Vid | N44°29'35.9'', E14°58'57.5'' | S. Wanke W085  (2004-04-08) | DR s.n. | OK019260 |
| *A. lutea* | P1413 | Croatia, Central Croatia, Varaždin County, Mt. Ravna Gora | N46°16'09.3", E15°59'15.4" | B. Oelschlägel, C. Pätzold  BO 2011-15 (2011-05-14) | DR040195 | OK019298 |
| *A. lutea* | P659 | Croatia, border of Primorje-Gorski Kotar County to Karlovac County, near Lokovo, Velika Kapela, east of Zagradski vrh. 0754/4 |  | K. Lewe Johan  CR-06-046 (2006-05-18) | GOET | OK019296 |
| *A. lutea* | P1414 | Croatia, Central Croatia, Krapina-Zagorje County, Strahinje | N46°10'43.8", E15°53'15.1" | B. Oelschlägel, C. Pätzold  BO 2011-16 (2011-05-16) | DR040196 | OK019299 |
| *A. lutea* | P183 | Croatia, Istria County, Brest, Mt. Žbevnica | N45°27'14.6'', E14°01'53.3'' | S. Wanke W 073  (2004-04-03) | DR | OK019286 |
| *A. lutea* | P182 | Croatia, Primorje-Gorski Kotar County, Breza | N45°25'24.1'', E14°20'22.4'' | S. Wanke W 089  (2004-04-10) | DR052213 | OK019285 |
| *A. lutea* | P658 | Croatia, Primorje-Gorski Kotar County, Crikvenica, near Gradina Badanj. 0854/1 |  | K. Lewe Johan CR-06-019 (2006-05-17) | GOET | OK019295 |
| *A. lutea* | P1416 | Croatia, Primorje-Gorski Kotar County, Klana | N45°26'49.2", E14°22'41.9" | B. Oelschlägel, C. Pätzold  BO 2011-19 (2011-05-18) | DR040199 | OK019300 |
| *A. lutea* | P210A | Croatia, Primorje-Gorski Kotar County, Lipa | N45°27'58.2'', E14°18'57.7'' | S. Wanke W 092  (2004-04-10) | DR052184 | OK019292 |
| *A. lutea* | P1417 | Croatia, Primorje-Gorski Kotar County, Lipa | N45°27'16.4", E14°18'54.0" | B. Oelschlägel, C. Pätzold  BO 2011-21 (2011-05-20) | DR040202 | OK019301 |
| *A. lutea* | P80 | Croatia, Primorje-Gorski Kotar County, Permani | N45°24'59.0'', E14°14'31.8'' | S. Wanke W 094  (2004-04-11) | DR | OK019276 |
| *A. lutea* | P66 | Croatia, Primorje-Gorski Kotar County, Poljane | N45°19'36.7'', E14°16'01.6'' | S. Wanke W 074  (2004-04-04) | DR | OK019275 |
| *A. lutea* | P217A | Croatia, Primorje-Gorski Kotar County, Škalnica | N45°26'41.4'', E14°21'09.1'' | S. Wanke W 091  (2004-04-10) | DR052183 | OK019293 |
| *A. lutea* | P186 | Croatia, Primorje-Gorski Kotar County, Studena | N45°25'00.8'', E14°23'41.0'' | S. Wanke W 090  (2005-04-10) | DR052212 | OK019288 |
| *A. lutea* | P185 | Croatia, Primorje-Gorski Kotar County, Veprinac | N45°21'32.5'', E14°16'59.1'' | S. Wanke W 093  (2004-04-11) | DR | OK019287 |
| *A. lutea* | 5 | Greece, Central Macedonia, Thessaloniki, Nea Madytos | N40°36'51.7'', E23°34'58.7'' | D. Frank, G. Lecocq A501 | STU SMNS-STU-PH-0099628 | MK095591 |
| *A. cf. lutea* | 1 | Greece, Thessalía, Karditsa, Kallifoni | N39°17'02.2'', E21°57'00.9'' | D. Frank, G. Lecocq A495 | STU SMNS-STU-PH-0099618 | MK095590 |
| *A. lutea* | 8 | Greece, Thessaly, Trikala, Koniskos |  | D. Frank, G. Lecocq A496 | STU SMNS-STU-PH-0099621 | MK095602 |
| *A. lutea* | P169 | Italy, Basilikata, Lago di Monticchio Piccolo | N40°56'13.2'', E15°37'02.1'' | S. Wanke, M. Neumann  W 190 (2005-05-15) | DR052167, DR052168, DR052169, DR052178 | OK019281 |
| *A. lutea* | P170 | Italy, Basilikata, Lago di Monticchio Piccolo | N40°56'13.2'', E15°37'02.1'' | S. Wanke, M. Neumann  W 195 (2005-05-15) | DR052177 | OK019282 |
| *A. lutea* | P173 | Italy, Basilikata, Potenza, Mt. Serranetta | N40°32'43.4'', E15°48'16.4'' | S. Wanke, M. Neumann  W 194 (2005-05-16) | DR052171, DR052172 | OK019283 |
| *A. lutea* | P168 | Italy, Basilikata, Rionero in Vulture, Mt. Vulture | N40°56'56.6'', E15°38'42.7'' | S. Wanke, M. Neumann  W 207 (2005-05-16) | DR052166, DR052170 | OK019280 |
| *A. lutea* | P174 | Italy, Calabria, Sant'Eufemia d'Aspromonte | N38°15'16.0'', E15°51'03.9'' | S. Wanke, M. Neumann  W 206 (2005-05-18) | DR052203, DR052210, DR052211 | OK019284 |
| *A. lutea* | 23 | Italy, Friuli-Venezia Giulia, Cividale del Friuli |  | N. Parrino A71 | STU SMNS-STU-PH-0100926 | MK095594 |
| *A. lutea* | P188 | Italy, Friuli-Venezia Giulia, Pulfero | N46°11'00.9'', E13°28'45.8'' | S. Wanke W 097  (2004-04-12) | DR052217, DR052224 | OK019290 |
| *A. lutea* | P218 | Italy, Friuli-Venezia Giulia, Repen | N45°43'37.1'', E13°46'18.3'' | S. Wanke W 096  (2004-04-12) | DR | OK019294 |
| *A. lutea* | P131 | Italy, Liguria, Varazze | N44°22'07.4'', E08°35'44.8'' | S. Wanke W 131  (2004-06-09) | DR | OK019277 |
| *A. lutea* | P132 | Italy, Toscany, Pescia | N43°55'19.3'', E10°44'07.4'' | S. Wanke W 134  (2004-06-11) | DR | OK019278 |
| *A. lutea* | P133 | Italy, Tuscany, Ruosina | N44°00'35.1'', E10°15'48.3'' | S. Wanke W 135  (2004-06-11) | DR | OK019279 |
| *A. lutea* | P189 | Italy, Veneto, Passo di san Boldo | N45°59'22.8'', E12°10'22.3'' | S. Wanke W 100  (2004-04-13) | DR052216 | OK019291 |
| *A. lutea* | P187 | Italy, Veneto, Quero | N45°56'01.3'', E11°54'45.3'' | S. Wanke W 102  (2004-04-13) | DR052218 | OK019289 |
| *A. lutea* | P1412 | Italy, Veneto, Quero | N45°56'17.8'', E11°54'47.8'' | B. Oelschlägel, C. Pätzold  BO 2011-1 (2011-04-29) | DR040191 | OK019297 |
| *A. lutea* | P60 | Slovenia, Coastal-Karst, Sežana | N45°42'06.4'', E13°51'48.6'' | S. Wanke W 095  (2004-04-12) | DR052226, DR052227, DR052228 | DQ296670 |
| *A. cf. lutea* | 20 | Turkey, Province Bolu |  |  | no voucher | MK095592 |
| *A. merxmuelleri* | 16 | Albania, Qarku Kukës, Kolsh |  | L. Shuka A411 | Tirana University | MK095596 |
| *A. merxmuelleri* | P158 | Kosovo, Mirusa, Konznik |  | Mayer E. 10.4.1968 | LJU, M 63193 (paratype) | DQ296673 |
| *A. microstoma* | P177 | Greece, Aegina island, Ag. Marina | N37°44'08.6'', E23°32'22.6'' | S. Wanke W 164  (2005-04-02) | DR052208 | OK019304 |
| *A. microstoma* | P178 | Greece, Attica, Mt. Parnitha | N38°08'12.7'', E23°44'01.8'' | S. Wanke W 183  (2005-03-24) | DR | OK019305 |
| *A. microstoma* | 2 | Greece, Central Greece, Boeotia, Arachova |  | D. Frank, G. Lecocq A492 | STU SMNS-STU-PH-0099624 | MK095597 |
| *A. microstoma* | P175 | Greece, Central Greece, Boeotia, Arachova | N38°28'54.4'', E22°37'13.6'' | S. Wanke W 175  (2005-03-31) | DR052206 | OK019302 |
| *A. microstoma* | P176 | Greece, Central Greece, Boeotia, Distomo | N38°26'01.0'', E22°39'17.4'' | S. Wanke W 174  (2005-03-31) | DR052236 | OK019303 |
| *A. microstoma* | P190 | Greece, Euböa island, Melissonas, Mt. Ochie | N38°05'29.0'', E24°23'59.9'' | S. Wanke W 167  (2005-03-31) | DR052189 | OK019308 |
| *A. microstoma* | P232 | Greece, Mt. Parnassos, plateau of Livadhi, 1067-1220m |  | Polunin 10227  (1970-05-29) |  | OK019309 |
| *A. microstoma* | P180 | Greece, Peloponnese, Argolis, Palea Epidauros | N37°38'29.4'', E23°09'54.2'' | S. Wanke W 171  (2005-03-25) | DR | OK019307 |
| *A. microstoma* | P135 | Greece, Peloponnese, Argolis, Palea Epidauros |  | M. Neumann 008 | DR025373 | DQ296672 |
| *A. microstoma* | P179 | Greece, Peloponnese, Argolis, Tolo | N37°30'55.9'', E22°51'30.1'' | S. Wanke W 179  (2005-03-25) | DR | OK019306 |
| *A. nardiana* | P146 | Greece, Peloponnes, Elis, Nea Figaleia | N37°25'58.0'', E21°48'17.9'' | S. Wanke W 170  (2005-03-28) | DR | OK019262 |
| *A. nardiana* | P148 | Greece, Peloponnes, Messenia, Langarda Pass | N37°04'45.0'', E22°16'52.4'' | S. Wanke W 162  (2005-03-27) | DR | OK019264 |
| *A. nardiana* | P150 | Greece, Peloponnes, Messenia, Mt. Kiparissias | N37°10'07.2'', E21°41'15.4'' | S. Wanke W 169  (2005-03-28) | DR | DQ296671 |
| *A. nardiana* | 6 | Greece, Stereá Elláda, Isle of Euboea, Pagontas |  | D. Frank, G. Lecocq A494 | STU SMNS-STU-PH-0099620 | MK095598 |
| *A. nardiana* | P149 | Greece, Thessaly, Karditsa, Loutropigi | N39°09'34.5'', E22°02'54.2'' | S. Wanke W 176  (2005-03-30) | DR | OK019265 |
| *A. nardiana* | P1236 | Greece, Thessaly, Trikala, Koniskos | N39°47'49'', E21°49'15'' | R. Eisenblätter, E.Willing 81.673 (2000-04-29) | B 10 0256257 | OK019268 |
| *A. nardiana* | P1235 | Greece, West Greece, Aetolia-Acarnania, Monastiraki | N38°50', E20°55'30'' | E. Willing 12.843  (1991-05-23) | B 10 0256258 | OK019267 |
| *A. nardiana* | P147 | Greece, West Greece, Aetolia-Acarnania, Riza | N38°21'49.7'', E21°42'50.1'' | S. Wanke W 155  (2005-03-29) | DR | OK019263 |
| *A. nardiana* | P1233 | Greece, West Macedonia, Grevena, Deskati | N39°53'30'', E21°46' | R. Willing 28.902  (1993-05-28) / Det. E. Willing | B 10 0256335 | OK019266 |
| *A. nardiana* | P1238 | Greece, West Macedonia, Grevena, Dhafnero | N40°11'04'', E21°35'32'' | R. Willing, E.Willing 100.693 (2002-04-17) | B 10 0091692 | OK019269 |
|  |  |  |  |  |  |  |
| *A. pallida* | 3 | France, Provence-Alpes-Côte d’Azur, Collobrières | N43°14'05.64'', E06°21'39.42'' | D. Frank, G. Lecocq A56 | STU SMNS-STU-PH-0099619 | MK095599 |
| *A. pallida* | P191 | France, Provence-Alpes-Côte d’Azur, Montfort | N44°03'55.9'', E05°56'03.4'' | S. Wanke W 187  (2005-05-07) | DR052221 | OK019317 |
| *A. pallida* | 7 | France, Provence-Alpes-Côte d’Azur, Ollières | N43°31'30.9'', E05°48'20.59'' | D. Frank, G. Lecocq A383 | STU SMNS-STU-PH-0099630 | MK095601 |
| *A. cf. pallida* | 4 | Greece, West Macedonia, Kozani, Dafnero |  | D. Frank, G. Lecocq A499 | STU SMNS-STU-PH-0099622 | MK095600 |
| *A. pallida* | P171 | Italy, Basilikata, Potenza, Monte Li Foie | N40°40'06.4'', E15°43'45.4'' | S. Wanke, M. Neumann  W 204 (2005-05-16) | DR052175, DR052173 | OK019314 |
| *A. pallida* | P172 | Italy, Basilikata, Potenza, Monte Li Foie | N40°40'03.6'', E15°43'35.3'' | S. Wanke, M. Neumann  W 196 (2005-05-16) | DR052174 | OK019315 |
| *A. pallida* | P134 | Italy, Liguria, Alpicella | N44°24'24.7'', E08°32'05.7'' | S. Wanke W 129  (2004-06-09) | DR052232 | OK019313 |
| *A. pallida* | P129 | Italy, Piedmont, Celle | N45°07'53.0'', E07°21'08.0'' | S. Wanke W 137  (2004-06-12) | DR052223 | OK019311 |
| *A. pallida* | P130 | Italy, Piedmont, Trana | N45°01'46.6'', E07°24'24.7'' | S. Wanke W 136  (2004-06-12) | DR052234 | OK019312 |
| *A. pallida* | P79 | Italy, Veneto, Guietta | N45°55'30.0'', E12°03'44.5'' | S. Wanke W 101  (2004-04-13) | DR052225 | DQ296669 |
| *A. pallida* | P184 | Italy, Veneto, Tarzo | N45°57'30.2'', E12°13'41.7'' | S. Wanke W 099  (2005-04-13) | DR052214, DR052215, DR052219, DR052222 | OK019316 |
| *A. pallida* | P978 | Turkey, Eskişehir to Söǧüt |  |  |  | OK019319 |
| *A. pallida* | P1220 | Turkey, Province Balıkesir, Sındırgı to Simav, 2km before intersection to Mumcuköy | N39°11’09”, E28°31’24” | A. Tosunoğlu, H.Malyer (2009-05-15) | BULU - AB. 1414/2 | OK019320 |
| *A. pallida* | P1227 | Turkey, Province Bursa, Bursa-Keles, kilometer 41 | N40°01’16’’, E29°07’15’’ | A. Bilişik, H.Malyer  (2008-05-01) | BULU - AB. 1119/2 | OK019327 |
| *A. pallida* | P1228 | Turkey, Province Bursa, Çalı to İnegazi, 200m bevor intersection to İnegazi | N40°08’03”, E28°52’53” | A. Tosunoğlu, H.Malyer (2009-05-13) | BULU - AB. 1401/3 | OK019328 |
| *A. pallida* | P1229 | Turkey, Province Bursa, Hüseyinalan | N40°06’51”, E29°01’20” | A. Tosunoğlu, H.Malyer (2009-05-13) | BULU - AB. 1404/3 | OK019329 |
| *A. pallida* | P976 | Turkey, Province Bursa, Nilüfer |  | A. Bilisik, H. Malyer  (2007-06-19) | Bulu,29055 | OK019318 |
| *A. pallida* | P1221 | Turkey, Province Çanakkale, Evciler | N39°45’51”, E26°46’48” | A. Tosunoğlu, H.Malyer (2009-05-14) | BULU - AB. 1408/4 | OK019321 |
| *A. pallida* | P1226 | Turkey, Province Eskişehir, south of Sarıcakaya, Avlakkaya | N39°57’23’’, E30°36’57’’ | A. Bilişik, H.Malyer  (2009-04-29) | BULU - AB. 1111/1 | OK019326 |
| *A. pallida* | P1225 | Turkey, Province Kütahya, Tavşanlı to Emet, 1km before intersection to Balıköy | N39°30’40’’, E29°23’58’’ | A. Bilişik, H.Malyer  (2008-05-03) | BULU - AB. 1128/1 | OK019325 |
| *A. pallida* | P1224 | Turkey, Province Kütahya, Tunçbilek | N39°43’17”, E29°30’58” | A. Tosunoğlu, H.Malyer (2008-05-03) | BULU - AB. 1126/4 | OK019324 |
| *A. pallida* | P1223 | Turkey, Province Manisa, Çukurhamami | N38°26’59”, E28°03’55” | A. Tosunoğlu, H.Malyer (2009-04-20) | BULU - AB. 1349/6 | OK019323 |
| *A. pallida* | P1222 | Turkey, Zonguldak Province, Devrek to Özbaği, 1km before the intersection to Sabunlar | N41°08’09”, E31°53’26” | A. Tosunoğlu, H.Malyer (2009-05-07) | BULU - AB. 1400/3 | OK019322 |
| *A. spec.* | P234 | Greece, Thessaly, Karditsa, Mt. Katacholoro |  | Stamatiadou 21583  (1980-05-08) | ATH | OK019337 |
|  |  |  |  |  |  |  |
| **Outgroup** |  |  |  |  |  |  |
| *A. acuminata* | 144A | BG Dresden, s.n. |  | Wanke & Neinhuis 146 | DR | DQ296646 |
| *A. albida* | 219A | BG Bonn, 17419 |  | Neinhuis 092 | DR | DQ296648 |
| *A. baetica* | 139A | BG Bonn, 14517 |  | Neinhuis 095 (DR) |  | DQ296653 |
| *A. bianorii* | 19 |  |  |  | no voucher | MK095588 |
| *A. bianorii* | P24 | Spain, Majorca, Betlem |  | S. Wanke 034 (DR) |  | DQ296664 |
| *A. bottae* | SW1 | Turkey, BG Bonn 02790 |  | leg. Koenen; Wanke 042 (DR) |  | DQ296659 |
| *A. bracteolata* | 216A | BG Bonn 16714 |  | Neinhuis 94 (DR) |  | DQ296647 |
| *A. clematitis* | P142 | Croatia, Is Ilovik/Asinello |  | Starmühler (KL) |  | DQ296651 |
| *A. clusii* | P224 | Italy, Bernalda |  | S. Wanke 192 | DR | OK019259 |
| *A. clusii* | P48 | Italy, Sicily, Villa de Marchese |  | Wanke & Neinhuis 104 | DR | DQ296666 |
| *A. debilis* | P19 | BG Dresden |  | S. Wanke 118 | DR | OK019261 |
| *A. eriantha* | 182A | BG Bonn 12952 |  | Neinhuis 99 | DR | DQ532054 |
| *A. fontanesii* | P62 | Algeria, Algier |  | leg. Abdelkrim; Wanke & Neinhuis 123 | DR | DQ296663 |
| *A. foveolata* | P123 |  |  | Murata et al. SETS4 | TI | OK019270 |
| *A. gaudichaudii* | P119 |  |  | Murata et al. SETS8 | TI | OK019271 |
| *A. guichardii* | P145 | Greece, Rhode, Asklepio |  | S. Wanke 186 | DR | OK019272 |
| *A. hirta* | P167 | Greece, Samos, Pirgos-Platanos |  | Neinhuis 134 | DR | DQ296657 |
| *A. iberica* | P107 | Georgia, Lagodechi National Park |  | leg. Gröger & Lobin 319-3; Wanke 210 | DR | DQ296655 |
| *A. incisa* | P163 | Greece, Samos, Pirgos-Platanos |  | Neinhuis 127 | DR | DQ296658 |
| *A. jackii* | P125 |  |  | Murata et al. SETS56 | TI | OK019273 |
| *A. kankauensis* | P124 |  |  | Murata et al. SETS35 | TI | OK019274 |
| *A. macrophylla* | 181A | BG Dresden, s.n. |  | Neinhuis s.n. | DR | DQ882193 |
| *A. navicularis* | P33 | Italy, Sardinia, Nora |  | S. Wanke W 021  (2003-04-19) | DR | OK019310 |
| *A. navicularis* | P43 | Italy, Sardinia, Donori |  | S. Wanke 019 | DR | DQ296661 |
| *A. parvifolia* | P214 | Greece, Rhode, Haraki |  | S. Wanke 177 | DR | OK019330 |
| *A. parvifolia* | P249 | Syria, Sallah Alden |  | Mafoud 033/2 | DR | OK019331 |
| *A. paucinervis* | P73 | BG Coimbra 135 |  | Wanke & Neinhuis 148 | DR | DQ296662 |
| *A. paucinervis* | PA1 | Spain |  | Andrea Costa |  | OK019332 |
| *A. pierrei* | P21 | Thailand s. loc. |  | s.coll, s.n. | DR | DQ296649 |
| *A. pistolochia* | P136 | Frankreich, Cassis, Calenque d'En Veau herb |  | H. Kreft; Wanke 037 | DR 025372 | DQ296652 |
| *A. pontica* | P139 | BG Bonn AC 4749 |  | S. Wanke 040 | DR | OK019333 |
| *A. rigida* | P83 | Somalia, Bulo Burti |  | Bally & Melville 15331 | K,MO | OK019334 |
| *A. rotunda* | P42 | France, Corsica, Figaretto Plage |  | Wanke 015 (2003-04-16) | DR | DQ296665 |
| *A. salvadorensis* | 08 | BG Bonn 10720 |  | Neinhuis 109 | DR | DQ882191 |
| *A. sempervirens* | P52 | Italy, Sicily, Avola |  | Wanke & Neinhuis 103 | DR | DQ296654 |
| *A. sempervirens* | P278 | Syria, Umaltueur |  | Mahfoud (HM 025/2) 08.04.2006 | DR | OK019335 |
| *A. sicula* | P155 | Italy, Sicily, Piano Zucchi |  | S. Wanke W 209  (2005-05-14) | DR | DQ296668 |
| *A. sicula* | P229 | Italy, Sicily, Piano Zucchi2 |  | S. Wanke 191 | DR | OK019336 |
| *A. tyrrhena* | P26 | Italy, Sardinia, San Nicolao |  | S. Wanke 024 | DR | DQ296667 |
| *A. tyrrhena* | P27 | France, Col de la Croix |  | S. Wanke 009 | DR | OK019338 |
| *A. westlandii* | 086A | BG Bonn 14211 |  | Neinhuis 115 | DR | DQ882192 |
